# Association between coagulation indicators and menorrhagia among women in Kenya

**DOI:** 10.4102/ajlm.v13i1.2438

**Published:** 2024-09-17

**Authors:** Phidelis M. Marabi, Stanslaus K. Musyoki, Fred Monari, Paul M. Kosiyo, Collins Ouma

**Affiliations:** 1 Department of Medical Laboratory Sciences, Faculty of Health Sciences, Kisii University, Kisii, Kenya; 2 Department of Biomedical Sciences, Faculty of Public Health and Community Development, Maseno University, Maseno, Kenya; 3 Department of Medical Laboratory Sciences, Faculty of Health Sciences, South Eastern Kenya University, Kitui, Kenya; 4 Department of Mathematics and Actuarial Science, Faculty of Pure Applied Sciences, Kisii University, Kisii, Kenya; 5 Department of Medical Laboratory Sciences, Faculty of Medicine, Maseno University, Maseno, Kenya

**Keywords:** menorrhagia, coagulation, profile, women, Kenya

## Abstract

**Background:**

Despite the significant burden of menorrhagia (bleeding > 80 mL every menstrual cycle) among women in Western Kenya, it remains unknown whether coagulation disorders are an important underlying cause of this condition in the region.

**Objective:**

This study assessed differences in coagulation profiles, associations between menorrhagia and coagulation profiles and compared morphological features of platelets among women attending Bungoma County Referral Hospital in Kenya.

**Methods:**

A comparative cross-sectional study of women with and without menorrhagia, aged 18–45 years, was performed between December 2022 and September 2023. Sociodemographic factors, prothrombin time (PT), activated partial thromboplastin time, thrombin time, fibrinogen, international normalised ratio (INR), and platelet count were compared between groups, and associations with menorrhagia were assessed. Prothrombin time and INR levels above normal references were deemed increased.

**Results:**

A total of 428 (214 per group) women were included. Family history of bleeding disorders (*p* < 0.0001) was more frequent in menorrhagic than in non-menorrhagic women. Additionally, menorrhagic women had high PT (*p* < 0.0001) and high INR (*p* < 0.0001) levels. Menorrhagia was significantly associated with an increased PT (odds ratio = 2.129, 95% confidence interval = 1.658–2.734; *p* < 0.0001) and increased INR (odds ratio = 7.479, 95% confidence interval = 3.094–18.080; *p* < 0.0001).

**Conclusion:**

In this population in Western Kenya, menorrhagia was associated with a family history of bleeding disorders, increased PT, and increased INR. Routine assessment of the coagulation profile and family history of bleeding disorders is crucial for diagnosing and managing menorrhagia.

**What this study adds:**

Our findings suggest that menorrhagic and non-menorrhagic women differ in terms of PT and INR, which may be predictive of menorrhagia.

## Introduction

Menorrhagia is defined as heavy menstrual bleeding that exceeds 80 mL per cycle.^1^ It is a common disorder that, on average, affects 30% of women.^2^ Having heavy periods significantly decreases women’s quality of life, requires time away from work, involves surgical intervention, including hysterectomy, and ultimately has a substantial cost impact on the healthcare system.^3^

Menorrhagia is a diagnosable ailment that needs to be treated to improve the quality of life of affected women,^4^ however, a significant number of menorrhagia cases are classified as idiopathic. Research has shown that when women are dissatisfied with their normal therapy options, they frequently choose surgery, such as hysterectomy.^5^ These women might benefit from a specialised method of diagnosis and treatment that emphasises pertinent coagulation parameters. A series of biochemical processes, known as the coagulation pathway, results in hemostasis.^6^ This complex pathway promotes healing and prevents spontaneous bleeding.^7^

An international expert panel in obstetrics, gynaecology, and haematology made recommendations on the best ways to manage menorrhagia in women without a known bleeding disorder.^8^ The proposals call for a laboratory assessment of complete blood counts, platelet functions, and coagulation profiles in menorrhagia patients; however, more than 61% of patients presenting with menorrhagia are still categorised as having a non-structural disorder without discernible coagulopathy as the cause of their condition.^9^ Menorrhagia may be caused by haemostatic disorders in as many as 17% – 20% of cases, according to a recent study in the United States (US).^10^ Nonetheless, coagulopathies are infrequently regarded as the cause of menorrhagia. A comprehensive history and clinical suspicion of an underlying bleeding issue in women who present with menorrhagia can help with the early identification and effective management of antepartum/postpartum haemorrhage.^11^ If there is no evident cause for menorrhagia, the coagulation profiles of all women should be checked. Coagulation problems are relatively common – 1% of the general population is affected, and such problems can affect as many as 5% of gynaecological patients.^12^ According to multiple studies conducted in high income countries, von Willebrand disease is the most common hereditary blood disorder that causes menorrhagia.^13^ In addition, previous research has shown that platelet function disorders are a major cause of heavy menstrual bleeding in adolescents.^14^ We hypothesise that derangements in coagulation profiles that affect the integrity of the intrinsic, extrinsic and common pathways in the coagulation cascade may be associated with menorrhagia. The explanations for coagulation problems and platelet dysfunction, which are some of the underlying causes of menorrhagia in women, are not well understood in Kenya. Furthermore, despite the substantial burden of menorrhagia (35.3%) in Western Kenya,^15^ and menorrhagia affecting approximately 3% of all gynaecological patients who visit the Bungoma County Referral Hospital (BCRH) clinic,^16^ the basic coagulation parameters of menorrhagic women compared to non-menorrhagic women are unknown. Therefore, this study set out to determine the differences in selected coagulation parameters between menorrhagic and non-menorrhagic women. In addition, the study investigated the associations between coagulation parameters and menorrhagia and the morphological characteristics of platelets from menorrhagic and non-menorrhagic women at BCRH, Kenya.

## Methods

### Ethical considerations

The study was approved by the Maseno University Scientific and Ethical Review Committee (reference number MUSERC/01166/22), and the authority to carry out the research was granted by the National Commission for Science, Technology, and Innovation (reference number NACOSTI/P/22/22573). Bungoma County Referral Hospital (reference number BDH/TP/B/VOL2) provided permission to perform the study. Each participant provided written informed consent after a brief explanation of the study. Measures taken to secure data included a password on the computer used and protected physical space. Protocol numbers were assigned, and personal information was collected from the participants in a confidential manner and utilised solely for the purpose of the study.

### Study area

The study was conducted at BCRH in Bungoma County, in Western Kenya, between December 2022 and September 2023. Bungoma County Referral Hospital is a level 5 hospital located within the headquarters of Bungoma County and is the main referral health facility for Bungoma County and its soundings, serving a population of 1 670 570 across an area of 2206.9 km^2^.^17^ Every year, approximately 6000 patients visit the BCRH gynaecological clinic.

### Study design and population

This study used a comparative cross-sectional design. The sample size formula for a quantitative comparative survey^18^ was used to calculate the number of study participants. The sample size was 428 (214 per group). A systematic random sampling procedure was adopted, with every second person satisfying the inclusion criteria being selected until the sample size was reached.

### Inclusion criteria

The study recruited adult women between the reproductive ages of 18 years and 45 years. The study included women who visited the clinic with complaints of menorrhagia. The comparison group consisted of healthy women picked at random. A qualified gynaecologist determined menorrhagia in each patient and established the clinical diagnosis based on the definition of menorrhagia as menstruation at regular cycle intervals but with excess flow (more than 80 mL of blood loss per cycle or requiring more frequent than 2 h changes of hygiene products) and/or duration (> 7 days).^19^

### Exclusion criteria

Women who experienced menorrhagia due to known causes, such as uterine fibroids or intrauterine contraception, were excluded from the study. Women with other known disorders that may alter coagulation parameters were excluded. Women who were found to be using any anticoagulants, as determined by a thorough clinical history by qualified clinicians, were also ineligible. Patients who had taken sylate or tranexamic acid within the previous 3 months, as well as other drugs known to manage bleeding difficulties, were ineligible.

### Sample and data collection

Using a screening procedure as used before,^20^ participants were classified into menorrhagic and non-menorrhagic women. A structured questionnaire was used to collect sociodemographic and clinical information, such as age, residence, parity, occupation, literacy, family history of bleeding disorders, history of epistaxis or gum bleeding, bleeding complications after tooth extraction, postoperative extended bleeding, and prolonged bleeding after delivery or miscarriage.

The phlebotomy order of drawing blood using evacuated tubes was blood collection in sodium citrate, followed by blood collection in ethylenediaminetetraacetic acid. For the coagulation profile, whole blood was collected from the participant’s arm by a qualified phlebotomist and put in a vacuum tube containing 3.2% sodium citrate (Becton, Dickinson and Company Corporate, Franklin Lakes, New Jersey, US), to ensure a 9:1 dilution of blood to the anticoagulant for prothrombin time (PT), international normalised ratio (INR), activated partial thromboplastin time (aPTT), thrombin time (TT) and fibrinogen tests. Thereafter, the participant’s distinctive identifying number tube was affixed to the tube. The sample(s) were delivered to the laboratory immediately after collection and spun for 5 min at 4000 rpm to ensure that the samples were platelet poor within 4 h. The separated samples were stored at 2°C – 8°C for 24 h if not tested immediately. The lyophilised coagulation test control materials (HUMAN Gesellschaft fur Biochemica and Diagnostica mbH, Wiesbaden, Germany) were reconstituted following the manufacturer’s instructions. For platelets count and morphology, whole blood was collected in a vacuum tube with ethylenediaminetetraacetic acid (Becton, Dickinson and Company Corporate, Franklin Lakes, New Jersey, US), immediately inverted gently 8–9 times, and labeled with a unique study number. Thin blood films for performing reflex testing when the analyser results were flagged and determining the platelet cell morphology were immediately prepared and fixed using absolute ethanol (Sigma-Aldrich, St. Louis, Missouri, US). The lyophilised control materials (HUMAN Gesellschaft fur Biochemica and Diagnostica mbH, Wiesbaden, Germany) were brought to room temperature and mixed well according to the manufacturer’s instructions before testing.

### Laboratory analyses

Coagulation profile parameters, including PT, aPTT, TT, fibrinogen, and the INR, were tested using a Humaclot Junior semiautomated coagulation analyser (HUMAN Gesellschaft fur Biochemica and Diagnostica mbH, Wiesbaden, Germany) as used previously.^21^ The analyser uses the photometric principle in which fibrin increases sample turbidity, which the photometer detects. After mixing the reagents with plasma, fibrinogen is converted into fibrin and coagulates. Thus, the optical density of the test sample changes, and the analyser can detect the coagulation endpoint.^22^ The fibrinogen assay was a quantitative clot-based test that assessed the rate of fibrinogen to fibrin conversion in a diluted sample in the presence of excess thrombin. Because the fibrinogen content is rate-limiting under these conditions, the clotting time is utilised to assess the concentration of the fibrinogen.^23^

For interpretation purposes, the reference ranges for the coagulation profile of adult women were adopted from the BCRH Laboratory as follows: PT: 11.0–12.5 s; aPTT: 30–40 s; TT: 15–19 s; fibrinogen: 200–400 mg/dL; and INR: 1.1 or less. The samples were examined at the same time as both abnormal and normal control plasma.

A Celtac F, MEK-8222 K analyser (NIHON KOHDEN Corporation, Tokyo, Japan), as described previously,^24^ was used to determine the thrombocyte count. This analyser measures thrombocytes by the electrical resistance sensing technique. For interpretation purposes, the reference ranges for the platelet count of adult women were adopted from the BCRH Laboratory as follows: 150–450 × 10^9^/L. Low, normal, and high levels of control materials were analysed before the samples were tested.

The morphology of the thrombocytes was evaluated using Leishman-stained blood films and Olympus^TM^ optical light compound microscopy (Olympus Global, Tokyo, Japan), as described previously,^25^ and the size, morphology, and granulation of the platelets were evaluated by a qualified medical laboratory technologist.

### Data analysis

Microsoft Excel sheets (Microsoft Corporation, Redmond, Washington, US) were used to store the data. The Statistical Package for Social Science (SPSS V.26) (IBM Corporation, Armonk, New York, US) was used for the analysis. The Shapiro-Wilk test was used to check for normality, and histograms and box plots were generated to verify the normality of the distribution of the data. The chi-square test was used to analyse categorical descriptive data that included residence, parity, occupation, family history of bleeding disorders, history of bleeding or gum bleeding, history of extended bleeding following tooth extraction, postoperative extended bleeding and history of prolonged bleeding following delivery or miscarriage. The Mann‒Whitney *U* test was used to compare coagulation profiles between menorrhagic and non-menorrhagic women, as the data were not normally distributed. Since the current study included a categorical dependent variable, binary logistic regression was used to predict the association between menorrhagia and coagulation parameters. The platelet morphological characteristics are presented as frequencies and percentages. The frequency, median, and dispersion of the descriptive statistics were shown. For all analyses, statistical significance was established at *p* ≤ 0.05.

## Results

### Demographic characteristics of the study population

A total of 428 adult women between the ages of 18 years and 45 years (median [Interquartile range]: 33.5 [11.0] years) participated in the study (Table 1). There were 214 menorrhagic patients and an equal number of non-menorrhagic healthy women. Participants did not differ with respect to place of residence in urban or rural location (*p* = 0.139), parity (*p* = 0.390), occupation (*p* = 0.846), history of epistaxis or gum bleeding (*p* = 0.499), and history of prolonged bleeding following delivery or miscarriage (*p* = 0.123) (Table 1). Menorrhagic women (*n* = 12/214; 5.6%) were more likely than non-menorrhagic women (*n* = 0/214; 0%) to have a family history of bleeding disorders (*p* < 0.001) (Table 1).

**TABLE 1:** Demographic characteristics of the study participants at Bungoma County Referral Hospital, Kenya, between December 2022 and September 2023.

Demographic characteristic	Women with menorrhagia (cases, *N* = 214)				Women without menorrhagia (controls, *N* = 214)				*p*
	*n*	%	Median	IQR	*n*	%	Median	IQR	
**Age (years) †**	-	-	34.0	6.0	-	-	33.0	14.0	0.209
**Residence ‡**	-	-	-	-	-	-	-	-	0.139
Rural	136	63.6	-	-	120	56.0	-	-	-
Urban	78	36.4	-	-	94	44.0	-	-	-
**Parity ‡**	-	-	-	-	-	-	-	-	0.390
Ever had a child	177	82.7	-	-	169	79.0	-	-	-
Never had a child	37	17.3	-	-	45	21.0	-	-	-
**Occupation ‡**	-	-	-	-	-	-	-	-	0.846
Formally employed	102	47.7	-	-	99	46.3	-	-	-
Informally employed	112	52.3	-	-	115	53.7	-	-	-
**Literacy**									N/A
Literate	214	100.0	-	-	214	100.0	-	-	-
Not literate	Nil	Nil	-	-	Nil	Nil	-	-	-
**Family history of bleeding disorders ‡**	-	-	-	-	-	-	-	-	< 0.001*
Yes	12	5.6	-	-	Nil	Nil	-	-	-
No	202	94.4	-	-	214	100.0	-	-	-
**History of epistaxis or gum bleeding ‡**	-	-	-	-	-	-	-	-	0.499
Yes	2	0.9	-	-	Nil	Nil	-	-	-
No	212	99.1	-	-	214	100.0	-	-	-
**History of bleeding complications following tooth extraction**	-	-	-	-	-	-	-	-	N/A
Yes	Nil	Nil	-	-	Nil	Nil	-	-	-
No	214	100.0	-	-	214	100.0	-	-	-
**Postoperative extended bleeding**	-	-	-	-	-	-	-	-	N/A
Yes	Nil	Nil	-	-	Nil	Nil	-	-	-
No	214	100.0	-	-	214	100.0	-	-	-
**History of prolonged bleeding following delivery or miscarriage ‡**	-	-	-	-	-	-	-	-	0.123
Yes	4	1.9	-	-	Nil	Nil	-	-	-
No	210	98.1	-	-	214	100.0	-	-	-

Note: Age (years) for women with menorrhagia (median: 34.0; IQR: 6.0) and for women without menorrhagia (median: 33.0; IQR: 14.0). A family history of bleeding disorders was significantly greater in menorrhagic women than in non-menorrhagic women.

IQR, interquartile range; N/A, the *p*-value was not generated; Nil, no demographic characteristic was seen in any of the participants for either the menorrhagic or non-menorrhagic categories.

* , *p* ≤ 0.05 indicate statistical significance.

† , Statistical significance was determined using Mann–Whitney *U* test.

‡ , Statistical significance of the differences was determined using the Chi-square test.

### Comparison of coagulation profiles between menorrhagic and non-menorrhagic women

Menorrhagic women (*n* = 38/214; 17.8%) were more likely to have increased PT than non-menorrhagic women (*n* = 3/214; 1.4%) (*p* < 0.0001) (Table 2). Menorrhagic women (*n* = 22/214; 10.3%) were more likely to have increased INR than non-menorrhagic women (*n* = 2/214; 0.9%) (*p* < 0.0001) (Table 2). Both menorrhagic and non-menorrhagic women showed no difference in terms of aPTT (*p* = 0.106), TT (*p* = 0.176), fibrinogen (*p* = 0.082) and platelet count (*p* = 0.101) (Table 2).

**TABLE 2:** Comparison of coagulation profiles between menorrhagic and non-menorrhagic women, Bungoma County Referral, Kenya, between December 2022 and September 2023.

Parameter	Women with menorrhagia (cases, *N* = 214)				Women without menorrhagia (controls, *N* = 214)				*p*
	*n*	%	Median	IQR	*n*	%	Median	IQR	
**Prothrombin time (s)**	-	-	12.40	2.40	-	-	12.10	0.50	< 0.00001*
Normal reference range: 11.00–12.50	176	82.2	-	-	211	98.6	-	-	-
> 12.50	38	17.8	-	-	3	1.4	-	-	-
**International normalised ratio**	-	-	1.27	0.51	-	-	0.80	0.50	< 0.00001*
Normal reference range: 1.1 or lower	192	89.7	-	-	212	99.1	-	-	-
> 1.1	22	10.3	-	-	2	0.9	-	-	-
**Activated partial thromboplastin time (s)**	-	-	35.14	5.80	-	-	34.68	3.19	0.106
Normal reference range: 30–40	214	100.0	-	-	214	100.0	-	-	-
> 40	0	0.0	-	-	0	0.0	-	-	-
**Thrombin time (s)**	-	-	17.39	2.84	-	-	17.11	1.89	0.176
Normal reference range: 15–19	209	97.7	-	-	214	100.0	-	-	-
> 19	3	1.4	-	-	0	0.0	-	-	-
< 15	2	0.9	-	-	0	0.0	-	-	-
**Fibrinogen (ng/dL)**	-	-	309.67	81.44	-	-	311.92	91.32	0.082
Normal reference range: 200–400	214	100.0	-	-	212	99.1	-	-	-
> 400	0	0.0	-	-	2	0.9	-	-	-
< 200	0	0.0	-	-	0	0.0	-	-	-
**Platelets count (× 10^9^/L)**	-	-	358.00	75.00	-	-	366.00	79.25	0.101
Normal reference range: 150–450	210	98.1	-	-	214	100.0	-	-	-
< 150	4	1.9	-	-	0	0.0	-	-	-

Note: Statistical significance was determined using the Mann‒Whitney *U* test. Prothrombin time and the International Normalised Ratio were significantly greater in menorrhagic women than in non-menorrhagic women, but the activated partial thromboplastin time, thrombin time, fibrinogen and platelet count were comparable.

IQR, interquartile range.

* , *p* ≤ 0.05 indicates statistical significance.

### Association between coagulation profile and menorrhagia

Menorrhagia was associated with increased PT (odds ratio [OR] = 2.129, 95% confidence interval [CI] = 1.658 – 2.734; *p* < 0.0001) and increased INR (OR = 7.479, 95% CI = 3.094–18.080; *p* < 0.0001) (Table 3). Menorrhagia was not associated with aPTT (OR = 1.064, 95% CI = 0.976–1.161; *p* = 0.158), TT (OR = 1.129, 95% CI = 0.961–1.325; *p* = 0.139), fibrinogen (OR = 0.996, 95% CI = 0.992–1.000; *p* = 0.062), or platelet count (OR = 0.998, 95% CI = 0.994–1.002; *p* = 0.349) (Table 3).

**TABLE 3:** Association between menorrhagia and abnormal coagulation profile, Bungoma County Referral Hospital, Kenya, between December 2022 and September 2023.

Parameter	Participant category	No. of participants	OR†	95% CI†	*p*
**Prothrombin time (s)**					
11.00–12.50	Non-menorrhagic	214	Ref.	-	-
> 12.50	Menorrhagic	214	2.129	1.658–2.734	< 0.0001*
**International normalised ratio**					
1.1 or lower	Non-menorrhagic	214	Ref.	-	-
> 1.1	Menorrhagic	214	7.479	3.094–18.080	< 0.0001*
**Activated partial thromboplastin time (s)**					
30–40	Non-menorrhagic	214	Ref.	-	-
> 40	Menorrhagic	214	1.064	0.976–1.161	0.158
**Thrombin time (s)**					
15–19	Non-menorrhagic	214	Ref.	-	-
> 19	Menorrhagic	214	1.129	0.961–1.325	0.139
**Fibrinogen (ng/dL)**					
200–400	Non-menorrhagic	214	Ref.	-	-
< 200	Menorrhagic	214	0.996	0.992–1.000	0.062
**Platelet count (× 10^9^/L)**					
150–450	Non-menorrhagic	214	Ref.	-	-
< 150	Menorrhagic	214	0.998	0.994–1.002	0.349

Note: Women (*N* = 428) were classified based on the presence or absence of menorrhagia. Menorrhagia was associated with high prothrombin time and International Normalised Ratio, but not with activated partial thromboplastin time, thrombin time, fibrinogen or platelet count.

CI, confidence interval; OR, odds ratio; Ref., reference; No., number.

* , *p* ≤ 0.05 indicates statistical significance.

† , Binary logistic regression analysis was used to obtain OR and 95% CI.

### Comparison of morphological characteristics of platelets between menorrhagic and non-menorrhagic women

In both menorrhagic and non-menorrhagic women, there were no morphologic differences in the platelets.

## Discussion

In this study, menorrhagic women were more likely to have a family history of bleeding disorders than non-menorrhagic women. Menorrhagic women had statistically significant increases in PTs and INRs compared with non-menorrhagic women, and menorrhagia had a statistically significant association with increased PT and INR. The demographic characteristics of the study participants were comparable between the two groups in terms of age, place of residence, parity, occupation, and literacy; however, menorrhagic women had no family history of bleeding disorders, while 5.6% of the menorrhagic women had a bleeding history of bleeding disorders. Due to the age range in this study, the median age of 34 years for menorrhagic women compared to 33 years of non-menorrhagic women was higher than those obtained in previous studies conducted in Sudan, India and Turkey.^26,27,28^

The residential areas of menopausal women (rural or urban) were not substantially different from those of non-menorrhagic women in the present study, which was similar to the findings of a study performed in Tanzania and published in 2023.^29^ The current study found that the majority of menorrhagic women had previously had a child, but no statistically significant difference in parity between menorrhagic and non-menorrhagic women. The current study revealed that a considerable number of menorrhagic women were informally employed, and there was no statistically significant difference from the findings for non-menorrhagic women. These findings are consistent with research performed in Tanzania and published in 2023,^29^ which showed that 52.5% of menorrhagic women work as peasants.

The present study found that whereas menorrhagic women were more likely to have a family history of bleeding disorders, almost three times as many menorrhagic women did not have a family history of bleeding disorders. None of the non-menorrhagic women had a family history of bleeding disorders. These findings are consistent with studies performed in Egypt, published in March 2014,^30^ Sudan, published in December 2015,^26^ and Sweden, published in 2006.^31^ Previous studies have shown a substantial association between idiopathic menorrhagia and a family history of heavy menstrual bleeding, suggesting that familial menorrhagia is an inherited feature.^28^ A family history of spontaneous or induced bleeding may increase the possibility of congenital bleeding, but multiple drugs can increase the risk of bleeding.^32^ However, some previous studies have demonstrated that one-third of menstrual bleeding problem diagnoses have no documented family history and can be caused by a random gene mutation.^33^ The high number of menorrhagic women without a family history of bleeding disorders can be attributed to the fact that a diagnosis of bleeding problems can develop completely unexpectedly due to a genetic mutation, which still needs further exploration. In addition, some people may be unaware of a bleeding issue in their family if other relatives are undiagnosed or have different symptoms. In light of the present findings, it is recommended that a thorough assessment of the family history of bleeding in menorrhagic women be performed routinely. Furthermore, women in this category should be evaluated for the possibility of having a genetic mutation of the F5 gene which has been found to cause factor V Leiden deficiency.^34^

The present study revealed that a history of epistaxis or gum bleeding in menorrhagic women was comparable to that in non-menorrhagic women; however, 0.9% of menorrhagic women had a history of epistaxis or gum bleeding. Additionally, the study showed that the history of prolonged bleeding following delivery or miscarriage in menorrhagic women and non-menorrhagic women was comparable; however, 1.9% of menorrhagic women had a history of prolonged bleeding following delivery or miscarriage. These findings agree with studies performed in South Sudan, published in December 2015,^26^ and Turkey, published in June 2013,^28^ which demonstrated that some patients had a history of additional bleeding issues in addition to presenting with menorrhagia. These findings could be attributed to the fact that previous studies have suggested that familial menorrhagia is a hereditary characteristic.^28^ In light of the findings of the current study, a history of bleeding disorders other than menorrhagia, comprising epistaxis, gum bleeding, and severe bleeding after delivery or miscarriage, should be evaluated extensively in women presenting with menorrhagia.

In the current investigation, the PT and INR were considerably greater in menorrhagic women than in non-menorrhagic women and were associated with menorrhagia, but the aPTT was comparable between the two groups and was not associated with menorrhagia. This was consistent with the results of a research done in Sudan, published in December, 2015,^26^ which demonstrated higher PT and INR in menorrhagic women than in non-menorrhagic women. These findings, however, contradicted those of a study undertaken in India, published in January 2011,^27^ which revealed PT to be normal in all except one patient. These findings also contradicted a study conducted in the US, published in 2013,^14^ which showed that PT levels were comparable between menorrhagic and non-menorrhagic women. The prolonged PT and INR and their association with menorrhagia in the current study could be attributed to the fact that women in the mid-luteal phase are more hypercoagulable than women in the early follicular phase.^35^ Most of those previous investigations associated PT with adenomyosis,^36^ but not directly with menorrhagia. However, prolonged PT and normal aPTT may reflect vitamin K deficiency caused by malnutrition, biliary obstruction, malabsorption syndromes, antibiotic use, or liver disease, which lowers clotting factor synthesis.^37^ The PT test evaluates the extrinsic coagulation pathway (also known as the tissue factor pathway), which comprises factors VII, X, V, and II, while the aPTT evaluates the intrinsic pathway (also known as the amplification pathway or contact system), which consists of factors XII, XI, IX, VIII, X, V, and II.^38^ It is further postulated that if the aPTT is normal, then a prolonged PT could be due to factor VII deficiency.^38^ In light of the present findings, it is recommended that PT and the INR, together with other coagulation factors, be routinely evaluated in menorrhagic women.

In the present study, TT, fibrinogen and platelet count were comparable in menorrhagic and non-menorrhagic women and were not associated with menorrhagia. Patients with dysfibrinogenaemia can also experience combined haemorrhagic and thrombotic tendencies.^39^ Dysfibrinogenaemia is a clotting disorder characterised by a range of structural defects in the fibrinogen molecule that result in aberrant fibrinogen function. It can be hereditary or acquired.^40^ In female patients with hereditary afibrinogenaemia, repeated severe intra-abdominal haemorrhages due to rupture of the Graafian follicle during ovulation have been reported.^41^ Patients with acquired dysfibrinogenaemia frequently have no history of bleeding or clotting, and a family history of dysfibrinogenaemia does not predict haematologic problems.^41^ Clinical symptoms of acquired dysfibrinogenaemia range from acute bleeding or thrombosis to chronic thromboembolic pulmonary hypertension and renal amyloidosis. Menorrhagia, postoperative bleeding, epistaxis, postoperative wound dehiscence, deficient wound healing, gingival bleeding, haemorrhage, and mild soft-tissue haemorrhage are all possible presenting signs and symptoms.^42^

Despite the current data indicating comparable platelet counts in menorrhagic and non- menorrhagic women, it is important to highlight that platelet count, size, and morphology are usually normal in individuals with Glanzmann thrombasthaenia, a hereditary qualitative condition that affects platelet function through failure of aggregation and others hereditary conditions.^43^ In the present study, neither large/giant platelets nor hyper-granular or agranular platelets were found in menorrhagic or non-menorrhagic women. Despite these findings, Bernard–Soulier syndrome can be linked to uncontrollable menorrhagia.^44^

Overall, the PT, INR, and family history of bleeding disorders, among other parameters, should be evaluated in menorrhagic women as soon as possible to better understand the coagulation profile and diagnose this life-threatening condition so that prompt treatment can be provided. A more accurate classification of menorrhagia would most likely result in the identification of a broader range of characteristics that could indicate haemostatic diseases or other aetiologies.

### Recommendations

The study recommends that women who experience heavy menstrual bleeding have a coagulation profile, as well as a family history of bleeding and history of bleeding disorders other than menorrhagia assessed. This approach allows for a more accurate diagnosis of menorrhagia based on the identified coagulation hallmark indicators.

### Limitations

The study was limited in that we did not assess von Willebrand factor, which is known to affect coagulation. Furthermore, the study did not perform platelet aggregometry as platelet function test to measure platelet response or aggregation to a panel of antagonists such as collagen, and did not measure vitamin K-dependent factors due to a lack of equipment at the laboratory and financial constraints. A longitudinal study with a larger sample size could help to close the knowledge gap in determining and interpreting such an association.

### Conclusion

In this study, a family history of bleeding disorders was present in some menorrhagic but not in non-menorrhagic women. Prothrombin time and INR were both increased among menorrhagic compared with non-menorrhagic women, and were associated with menorrhagia. Thus, family history of bleeding disorders, PT and the INR, which are coagulation parameters, may contribute to the aetiology of menorrhagia.
